# Genetic overlap between endometriosis and endometrial cancer: evidence from cross‐disease genetic correlation and GWAS meta‐analyses

**DOI:** 10.1002/cam4.1445

**Published:** 2018-04-02

**Authors:** Jodie N. Painter, Tracy A. O'Mara, Andrew P. Morris, Timothy H. T. Cheng, Maggie Gorman, Lynn Martin, Shirley Hodson, Angela Jones, Nicholas G. Martin, Scott Gordon, Anjali K. Henders, John Attia, Mark McEvoy, Elizabeth G. Holliday, Rodney J. Scott, Penelope M. Webb, Peter A. Fasching, Matthias W. Beckmann, Arif B. Ekici, Alexander Hein, Matthias Rübner, Per Hall, Kamila Czene, Thilo Dörk, Matthias Dürst, Peter Hillemanns, Ingo Runnebaum, Diether Lambrechts, Frederic Amant, Daniela Annibali, Jeroen Depreeuw, Adriaan Vanderstichele, Ellen L. Goode, Julie M. Cunningham, Sean C. Dowdy, Stacey J. Winham, Jone Trovik, Erling Hoivik, Henrica M. J. Werner, Camilla Krakstad, Katie Ashton, Geoffrey Otton, Tony Proietto, Emma Tham, Miriam Mints, Shahana Ahmed, Catherine S. Healey, Mitul Shah, Paul D. P. Pharoah, Alison M. Dunning, Joe Dennis, Manjeet K. Bolla, Kyriaki Michailidou, Qin Wang, Jonathan P. Tyrer, John L. Hopper, Julian Peto, Anthony J. Swerdlow, Barbara Burwinkel, Hermann Brenner, Alfons Meindl, Hiltrud Brauch, Annika Lindblom, Jenny Chang‐Claude, Fergus J. Couch, Graham G. Giles, Vessela N. Kristensen, Angela Cox, Krina T. Zondervan, Dale R. Nyholt, Stuart MacGregor, Grant W. Montgomery, Ian Tomlinson, Douglas F. Easton, Deborah J. Thompson, Amanda B. Spurdle

**Affiliations:** ^1^ Department of Genetics and Computational Biology QIMR Berghofer Medical Research Institute Brisbane Queensland Australia; ^2^ Wellcome Trust for Human Genetics University of Oxford Oxford UK; ^3^ Department of Biostatistics University of Liverpool Liverpool UK; ^4^ Wellcome Trust Centre for Human Genetics and Oxford NIHR Biomedical Research Centre University of Oxford Oxford UK; ^5^ Department of Clinical Genetics St George's, University of London London UK; ^6^ Institute for Molecular Bioscience University of Queensland Brisbane Queensland Australia; ^7^ Hunter Medical Research Institute John Hunter Hospital Newcastle New South Wales Australia; ^8^ Centre for Clinical Epidemiology and Biostatistics School of Medicine and Public Health University of Newcastle Callaghan New South Wales Australia; ^9^ Division of Molecular Medicine Pathology North, John Hunter Hospital Newcastle New South Wales Australia; ^10^ Discipline of Medical Genetics School of Biomedical Sciences and Pharmacy Faculty of Health University of Newcastle Callaghan New South Wales Australia; ^11^ QIMR Berghofer Medical Research Institute Brisbane Queensland Australia; ^12^ Department of Gynaecology and Obstetrics University Hospital Erlangen Comprehensive Cancer Center Erlangen‐EMN Friedrich‐Alexander University Erlangen‐Nuremberg Erlangen Germany; ^13^ David Geffen School of Medicine Department of Medicine Division of Hematology and Oncology University of California at Los Angeles Los Angeles California; ^14^ Institute of Human Genetics University Hospital Erlangen Comprehensive Cancer Center Erlangen‐EMN Friedrich‐Alexander University Erlangen‐Nuremberg Erlangen Germany; ^15^ Department of Medical Epidemiology and Biostatistics Karolinska Institutet Stockholm Sweden; ^16^ Gynaecology Research Unit Hannover Medical School Hannover Germany; ^17^ Department of Gynaecology Jena University Hospital ‐ Friedrich Schiller University Jena Germany; ^18^ VIB Center for Cancer Biology, VIB Leuven Belgium; ^19^ Laboratory for Translational Genetics Department of Human Genetics University of Leuven Leuven Belgium; ^20^ Department of Obstetrics and Gynecology Division of Gynecologic Oncology University Hospitals KU Leuven University of Leuven Leuven Belgium; ^21^ Vesalius Research Center, VIB Leuven Belgium; ^22^ Department of Health Sciences Research Mayo Clinic Rochester Minnesota; ^23^ Department of Laboratory Medicine and Pathology Mayo Clinic Rochester Minnesota; ^24^ Department of Obstetrics and Gynecology Division of Gynecologic Oncology Mayo Clinic Rochester Minnesota; ^25^ Centre for Cancerbiomarkers Department of Clinical Science University of Bergen Bergen Norway; ^26^ Department of Obstetrics and Gynecology Haukeland University Hospital Bergen Norway; ^27^ Centre for Information Based Medicine University of Newcastle Callaghan New South Wales Australia; ^28^ School of Medicine and Public Health University of Newcastle Callaghan New South Wales Australia; ^29^ Department of Molecular Medicine and Surgery Karolinska Institutet Stockholm Sweden; ^30^ Clinical Genetics Karolinska Institutet Stockholm Sweden; ^31^ Department of Women's and Children's Health Karolinska Institutet Stockholm Sweden; ^32^ Department of Oncology Centre for Cancer Genetic Epidemiology University of Cambridge Cambridge UK; ^33^ Department of Public Health and Primary Care Centre for Cancer Genetic Epidemiology University of Cambridge Cambridge UK; ^34^ Department of Electron Microscopy/Molecular Pathology The Cyprus Institute of Neurology and Genetics Nicosia Cyprus; ^35^ Centre for Epidemiology and Biostatistics Melbourne School of Population and Global Health The University of Melbourne Melbourne Victoria Australia; ^36^ Department of Non‐Communicable Disease Epidemiology London School of Hygiene and Tropical Medicine London UK; ^37^ Division of Genetics and Epidemiology The Institute of Cancer Research London UK; ^38^ Division of Breast Cancer Research The Institute of Cancer Research London UK; ^39^ Department of Obstetrics and Gynecology University of Heidelberg Heidelberg Germany; ^40^ Molecular Epidemiology Group, C080 German Cancer Research Center (DKFZ) Heidelberg Germany; ^41^ Division of Clinical Epidemiology and Aging Research German Cancer Research Center (DKFZ) Heidelberg Germany; ^42^ Division of Preventive Oncology German Cancer Research Center (DKFZ) National Center for Tumor Diseases (NCT) Heidelberg Germany; ^43^ German Cancer Consortium (DKTK) German Cancer Research Center (DKFZ) Heidelberg Germany; ^44^ Division of Gynaecology and Obstetrics Technische Universität München Munich Germany; ^45^ Dr. Margarete Fischer‐Bosch‐Institute of Clinical Pharmacology Stuttgart Germany; ^46^ University of Tübingen Tübingen Germany; ^47^ Division of Cancer Epidemiology German Cancer Research Center (DKFZ) Heidelberg Germany; ^48^ Research Group Genetic Cancer Epidemiology University Cancer Center Hamburg (UCCH) University Medical Center Hamburg‐Eppendorf Hamburg Germany; ^49^ Cancer Epidemiology & Intelligence Division Cancer Council Victoria Melbourne Victoria Australia; ^50^ Department of Cancer Genetics Institute for Cancer Research Oslo University Hospital Radiumhospitalet Oslo Norway; ^51^ Institute of Clinical Medicine Faculty of Medicine University of Oslo Oslo Norway; ^52^ Department of Clinical Molecular Biology Oslo University Hospital University of Oslo Oslo Norway; ^53^ Department of Oncology and Metabolism Sheffield Institute for Nucleic Acids (SInFoNiA) University of Sheffield Sheffield UK; ^54^ Department of Obstetrics & Gynaecology Endometriosis CaRe Centre, Nuffield University of Oxford Oxford UK; ^55^ Institute of Health and Biomedical Innovation and School of Biomedical Science Queensland University of Technology Brisbane Queensland Australia

**Keywords:** Cross‐disease analysis, endometrial cancer, endometriosis, genome‐wide association study, genetic correlation

## Abstract

Epidemiological, biological, and molecular data suggest links between endometriosis and endometrial cancer, with recent epidemiological studies providing evidence for an association between a previous diagnosis of endometriosis and risk of endometrial cancer. We used genetic data as an alternative approach to investigate shared biological etiology of these two diseases. Genetic correlation analysis of summary level statistics from genomewide association studies (GWAS) using LD Score regression revealed moderate but significant genetic correlation (*r*_g _= 0.23, *P *=* *9.3 × 10^−3^), and SNP effect concordance analysis provided evidence for significant SNP pleiotropy (*P *=* *6.0 × 10^−3^) and concordance in effect direction (*P *=* *2.0 × 10^−3^) between the two diseases. Cross‐disease GWAS meta‐analysis highlighted 13 distinct loci associated at *P *≤* *10^−5^ with both endometriosis and endometrial cancer, with one locus (SNP rs2475335) located within *PTPRD* associated at a genomewide significant level (*P *=* *4.9 × 10^−8^, OR = 1.11, 95% CI = 1.07–1.15). *PTPRD* acts in the STAT3 pathway, which has been implicated in both endometriosis and endometrial cancer. This study demonstrates the value of cross‐disease genetic analysis to support epidemiological observations and to identify biological pathways of relevance to multiple diseases.

## Introduction

Endometriosis (defined as tissue resembling endometrium in extrauterine sites) and endometrial cancer (cancer of the uterine corpus) are serious gynecological diseases with major impacts on the quality of life of affected women. Endometriosis is a relatively common disease affecting 6–10% of women of reproductive age and 35–50% of infertile women 1, 2. Affected women commonly experience severe menstrual pain, pelvic pain, subfertility or infertility, and bowel‐related symptoms. Endometrial cancer is the most common invasive gynecological cancer in Australia, ranking sixth for incident cancers in women 3. This disease is associated with significant morbidity due to surgery and radiotherapy 4, and treatment is further complicated by the fact that most patients present at relatively older age and with major comorbidities, notably obesity and diabetes. Finding the genes and pathways underlying these complex diseases is an essential step toward developing better diagnostic and therapeutic tools for both diseases. Both diseases are known to have a genetic component, with twin studies showing heritability of endometriosis at ~50% (51%, 95% confidence interval = 33–66% 5; *H* = 47%, 95% CI = 36–57% 6) and of endometrial cancer 27% (95% CI = 11–43% 7). Genomewide association studies have, to date, identified 19 independent SNPs as being significantly associated with endometriosis 8 and nine independent SNPs with endometrial cancer 9, 10. These genomewide‐associated SNPs, and the genetic regions in which they occur, are nonoverlapping between the diseases.

Epidemiological, biological, and molecular data all indirectly suggest that there could be links between the two disorders. Endometriosis and endometrial cancer are both hormonally regulated diseases, with increased risk in women exposed to higher levels of estrogen, and decreased or ameliorated risk or symptoms through treatments such as the contraceptive pill and hormonal therapies that include progesterone 11. Both are associated with increased risk of uterine fibroids 12, 13 and with ovarian cancer: endometriosis through an increased risk of this disease, and endometrial cancer through multiple shared risk factors, and histopathologic and molecular features 14, 15. Cancer‐related genetic changes such as loss of heterozygosity, and altered methylation and expression patterns have been reported for endometriosis 16. Numerous endometrial cancer‐associated genes, including *PTEN* and other genes in the Ingenuity “endometrial cancer pathway,” have been shown to be dysregulated in endometriosis 17, 18.

Epidemiological studies have shown conflicting evidence for a link between a diagnosis of endometriosis and risk of endometrial cancer 13, 19, 20, 21, 22, 23, 24. The interpretation of results from epidemiological studies is complicated by several factors, including small sample sizes, the underdiagnosis and misdiagnosis of endometriosis, inability to adjust for confounders including oral contraceptives and parity, and the exclusion criteria of some epidemiological studies which assumed coincidental diagnosis of endometrial cancer in women ascertained via a diagnosis of endometriosis. For example, Rowlands et al. showed an overall 1.5‐fold increased risk of endometrial cancer that was reduced by excluding cases diagnosed with endometriosis <1 year before the endometrial cancer diagnosis; however, the subset of women with *surgically confirmed* endometriosis diagnosed >1 year prior to cancer showed a significant 2.6‐fold increased risk of endometrial cancer 13. However, a recent study in US nurses which was also able to adjust for diagnosis intervals found no association between either self‐reported or laparoscopically confirmed endometriosis and risk of endometrial cancer 22. Meanwhile, two population‐based studies had shown associations between the diseases, although neither was able to adjust for confounders such as parity. A large study including 45,790 Danish women with a clinical diagnosis of endometriosis found increased risks of endometrial cancer >1 year (standardized incidence ratio (SIR) = 1.43, 95% CI = 1.13–1.79) and ≥10 years (SIR = 1.51, 95% CI = 1.15–1.95) following the endometriosis diagnosis 23. Another study including 15,488 Taiwanese women diagnosed with endometriosis found a similar link, but only in women diagnosed with endometriosis at over 40 years of age (adjusted hazard ratio = 7.08, 95% CI = 2.33–21.55) 24. Age‐related effects, if present, could have further confounded the results of previous epidemiological studies investigating shared risk of endometriosis and endometrial cancer.

Given the methodological complications inherent in epidemiological studies, unbiased genetic approaches are an ideal way to test for shared biological etiology between endometriosis and disease. For example, a degree of shared genetic etiology has recently been demonstrated between endometriosis and ovarian cancer, including with ovarian cancer subtypes not previously thought to be associated with endometriosis 25. We used separate genomewide association study (GWAS) datasets for endometriosis and endometrial cancer to estimate the degree to which these two diseases share a common genetic etiology. We then combined these datasets in a cross‐disease GWAS meta‐analysis to identify genetic loci potentially contributing to the genetic risk of *both* endometriosis and endometrial cancer.

## Materials and Methods

### Genetic overlap between endometriosis and endometrial cancer: datasets and analyses

This study utilized data from four previously published genetic datasets for endometriosis and endometrial cancer (outlined below and in the following section; Table 1) 26, 27, 28. Three of the datasets were GWAS datasets, genotyped using Illumina 610Quad and 670Quad BeadChips (Illumina Inc, San Diego, CA) and containing data for 462,430 SNPs in common between them. Of these, the endometriosis GWAS dataset included 3194 Australian (QIMR Berghofer Medical Research Institute (QIMR)) and UK (Oxford) women with surgically confirmed endometriosis as cases 26. The first endometrial cancer GWAS dataset included 1262 Australian (ANECS) and UK (SEARCH) endometrioid subtype endometrial cancer patients 27, and the second (NSECG) included 795 UK endometrial cancer cases and 895 nonoverlapping controls 28. All endometrial cancer cases were histologically confirmed to be invasive cancer of the endometrium lining 27. As previously published, the endometriosis and ANECS‐SEARCH endometrial cancer GWAS datasets included the same sets of controls—1870 Australian controls and 5190 UK Wellcome Trust Case Control Consortium (WTCCC) controls. Hence to avoid overlapping control samples in this study, the controls were redistributed as follows: The 1870 Australian controls and two‐third of the WTCCC controls (*n* = 3460, randomly assigned) were included in the endometriosis GWAS dataset, while an additional set of 1241 Australian controls 28 and the remaining one‐third of the WTCCC controls (*n* = 1730) were included in the ANECS‐SEARCH endometrial cancer GWAS dataset.

**Table 1:** Endometriosis and endometrial cancer datasets utilized in the genetic overlap and genomewide association meta‐analyses

Dataset^a^	Case *N*	Control *N*	Genotyping platform	Analysis: Genetic overlap	Meta‐analysis
Endometriosis					
QIMR	2270	1870	Illumina 660K	√	√
Oxford	924	3460	Illumina 660K	√	√
Endometrial cancer					
ANECS	591	1241	Illumina 610K	√	√
SEARCH	671	1730	Illumina 610K	√	√
NSECG	795	895	Illumina 660K	√	√
iCOGS	4402	28,758	Custom Illumina array	–	√

Further details of the endometrial cancer studies contributing to the various datasets are included in Table S1.

Following quality control 26, 27, 28, association analyses were performed for each GWAS dataset using PLINK 29. Australian and UK cases and controls were analyzed as separate strata within the same GWAS for the endometriosis and ANECS‐SEARCH endometrial cancer datasets, adjusting for the first two (endometriosis, ANECS, NSECG) or three (SEARCH) principal components of the genomic kinship matrix 26, 27, 28. The summary results for the ANECS‐SEARCH and NSECG datasets were then included in an inverse variance, fixed effects meta‐analysis performed using METAL 30, to produce one set of endometrial cancer GWAS results. A fixed effect model was considered more appropriate than a random effect model as our hypothesis is that a proportion of SNPs will be associated with both diseases with the same direction of effect, and a fixed effect model is conservative given no expectation that the effect size is similar. The degree of genetic overlap between endometriosis and endometrial cancer was then examined using two programs that test the degree of genetic correlation/concordance between diseases using GWAS summary results (individual SNP effect sizes and *P*‐values), SNP effect concordance analysis (SECA) 31 and LD Score regression 32.

To account for linkage disequilibrium (LD) between SNPs, SECA employs a “*P*‐value informed” SNP clumping procedure to extract a subset of independent SNPs 31 (23,817 SNPs for the current analysis). These SNPs are then partitioned into 12 *P*‐value “bins” (e.g., *P *≤* *0.01, 0.05, 0.1, 0.2, 0.3, 0.4, 0.5, 0.6, 0.7, 0.8, 0.9, and 1.0) for each disease. Using the default settings, a number of binomial and Fisher exact tests were performed on SNPs across all bins (12 × 12 bins = 144 SNP subset combinations), and on SNP subsets within bins (see Results), to determine the degree to which individual SNPs are concordant in their *P*‐value level and direction of effect across two diseases, which can indicate the presence of genetic concordance and SNP pleiotropy 31. For these analyses, the endometriosis dataset was designated as Dataset 1 and endometrial cancer as Dataset 2.

Taking a different approach, cross‐trait LD Score regression utilizes the presence of LD, calculating an LD score between SNPs within a 1 cM window and then regressing the product of the SNP association results (*z* scores) from the two diseases against the LD score 32. Following the recommendations at https://github.com/bulik/ldsc/wiki/Heritability-and-Genetic-Correlation, the “–no‐intercept” option was used to constrain the LD Score regression intercept to 0 as there was no sample overlap between the two disease datasets.

### Cross‐disease meta‐analysis between endometriosis and endometrial cancer

The cross‐disease meta‐analysis was performed using an inverse variance, fixed effects model in METAL 30 to search for genetic loci potentially contributing to the increased risk of both endometriosis and endometrial cancer. Heterogeneity was assessed using Cochran's *Q*‐test. The results for the top SNPs (*P *≤* *10^−5^) from the endometriosis–endometrial cancer meta‐analysis were then compared with results for the same SNPs from the fourth dataset included in this study, a separate, independent sample of 4402 endometrial cancer cases and 28,758 controls genotyped at 211,155 SNPs using a custom Illumina Infinium iSelect array by the Collaborative Oncological Gene‐environment Study (“iCOGS”) 33, 34. SNPs not included on the iCOGS array were imputed (including all SNPs within 1 Mb of the target SNP) using IMPUTE(v2) software 35 and the 1000 Genomes Project (2012 release) as the reference panel 9. Imputation quality scores ranged from 0.34 to 1.00. Association testing on the iCOGS SNPs was performed using SNPTEST (v2) 36 employing frequentist tests with a logistic regression model adjusting for eight separate strata and the first 10 principal components 9, 28. These results were then included in the replication meta‐analysis, which included all four datasets and was conducted as described for the cross‐disease meta‐analysis above.

## Results

### Genetic overlap between endometriosis and endometrial cancer

Genetic correlation analyses of GWAS datasets for endometriosis and endometrial cancer revealed the presence of weak to moderate, but significant, genetic overlap between the two diseases. The LD Score regression analysis indicated moderate but significant genetic correlation (r_g_) between the two diseases (*r*_g_ = 0.23, *P *=* *9.3 × 10^−3^). The SECA primary test for the overlap of associated effects, including all 144 SNP subsets, revealed more subsets than expected by chance showing at least nominally significant pleiotropy between endometriosis and endometrial cancer (*P *=* *6.0 × 10^−3^): The pair of SNP subsets producing the minimum exact binomial test *P*‐value for pleiotropy (endometriosis SNP subset with *P *≤* *0.002 and endometrial cancer SNP subset with *P *≤* *0.86) had *P *=* *3.3 × 10^−4^. The primary test for concordant effects between endometriosis and endometrial cancer also revealed that the number of SNP subsets with nominally significant concordant effects (*P *≤* *0.05) was significantly more than expected by chance (*P *=* *2.0 × 10^−3^): The pair of SNP subsets producing the minimum Fisher's exact test *P*‐value for effect correlation (endometriosis SNP subset with *P *≤* *0.37 and endometrial cancer SNP subset with *P* ≤ 1) had *P *=* *2.1 × 10^−4^. The primary results indicate that SNP effects are correlated, with the presence of allelic effects that increase the risk of both traits. Including only specific (default) SNP subsets in the analyses 31, SNP effects were positively, although not significantly, correlated for SNPs at *P *≤* *0.05 in both datasets (*P *=* *8.4 × 10^−2^) and for SNPs with *P *≤* *1.0 × 10^−5^ in the larger endometriosis dataset and with *P *≤* *0.05 in the endometrial cancer dataset (*P *=* *6.8 × 10^−2^). Together, these results indicate that overall more SNPs than expected by chance were associated with the same direction of effect for both diseases, particularly amongst nominally or marginally associated SNPs.

### Cross‐disease genomewide association analyses

For the cross‐disease meta‐analysis including the endometriosis and endometrial cancer (ANECS‐SEARCH and NSECG) GWAS datasets, two SNPs had *P*‐values reaching genomewide significance (rs6782972, *P *=* *3.3 × 10^−9^ and rs2218868, *P *=* *4.1 × 10^−8^; Table S1). A further 92 SNPs were suggestively (*P *≤* *10^−5^) associated in the combined analysis of both diseases (Table S1). Following inclusion of the iCOGs association results in the meta‐analysis, the *P*‐values for both rs6782972 and rs2218868 dropped below the threshold for suggestive significance (rs6782972, *P *=* *1.2 × 10^−2^, OR = 0.95, 95% CI = 0.90–0.99; rs2218868, *P *=* *1.4 × 10^−3^, OR = 1.06, 95% CI = 1.02–1.09); hence, neither locus was validated by the endometrial cancer replication dataset. Including all four datasets, 13 loci showed evidence for replication (*P*‐values ≤10^−5^; Table 2; Fig. S1), with a genomewide significant signal detected for SNP rs2475335 (*P *=* *4.9 × 10^−8^, OR = 1.11, 95% CI = 1.07–1.14). After adjusting for the multiple testing of 13 SNPs, two SNPs showed evidence of significant heterogeneity between studies (Table 2), rs9865110 (*P*_het_ = 2.2 × 10^−3^) and rs7515106(*P*_het_ = 3.6 × 10^−3^).

**Table 2:** Replicated associations (*P* ≤ 10^−5^) from the cross‐disease meta‐analysis for endometriosis and endometrial cancer

Chr	BP	Lead SNP	Flanking gene/genes^a^	Effect allele	Noneffect allele	GWAS meta‐analysis		Replication meta‐analysis		*P*_het_
						OR (95% CI)	*P*‐value	OR (95% CI)	*P*‐value	
9	10260263	rs2475335	*PTPRD*	T	C	1.15 (1.09–1.21)	2.1 × 10^−6^	1.11 (1.07–1.15)	4.9 × 10^−8^	0.46
3	73981773	rs9865110	*PDZRN3‐CNTN3*	C	A	1.11 (1.06–1.16)	9.4 × 10^−5^	1.10 (1.06–1.14)	2.6 × 10^−6^	0.002
17	46262171	rs2278868	*SKAP1*	C	T	0.87 (0.82–0.93)	4.7 × 10^−7^	0.92 (0.88–0.96)	5.5 × 10^−6^	0.05
12	89299016	rs12303900	*KITLG‐DUSP6*	G	T	1.30 (1.17–1.43)	9.7 × 10^−5^	1.28 (1.17–1.38)	5.7 × 10^−6^	0.08
6	50715407	rs9349553	*TFAP2D*	T	C	1.14 (1.08–1.19)	4.5 × 10^−6^	1.09 (1.05–1.13)	9.0 × 10^−6^	0.02
4	38765720	rs10008492	*KLF3‐TLR10*	T	C	1.14 (1.08–1.19)	2.3 × 10^−6^	1.08 (1.05–1.12)	1.3 × 10^−5^	0.07
13	77164473	rs9530566	*LMO7‐KCTD12*	C	A	1.12 (1.07–1.17)	3.3 × 10^−5^	1.08 (1.05–1.12)	1.7 × 10^−5^	0.054
12	4253697	rs10459129	*PARP11‐CCND2*	A	G	0.89 (0.83–0.95)	6.8 × 10^−5^	0.90 (0.86–0.95)	2.1 × 10^−5^	0.44
19	31364717	rs2198894	*ZNF536‐TSHZ3*	T	C	1.11 (1.06–1.16)	8.4 × 10^−5^	1.09 (1.05–1.13)	2.4 × 10^−5^	0.49
9	32907608	rs7042500	*THEM215‐APTX*	A	G	0.86 (0.79–0.93)	1.2 × 10^−5^	0.90 (0.86–0.95)	4.0 × 10^−5^	0.07
17	63665556	rs17693745	*CEP112*	T	C	1.12 (1.07–1.18)	2.3 × 10^−5^	1.08 (1.04–1.12)	5.3 × 10^−5^	0.24
1	22473410	rs7515106	*WNT4‐ZBTB40*	C	T	1.15 (1.08–1.21)	1.2 × 10^−5^	1.09 (1.04–1.14)	7.0 × 10^−5^	0.0036
6	57186294	rs1755833	*PRIM2*	A	G	0.89 (0.84–0.95)	3.9 × 10^−5^	0.93 (0.89–0.97)	7.9 × 10^−5^	0.007

a Only one gene is listed for SNPs located within genes (introns or exons).

## Discussion

A link between endometriosis and endometrial cancer has long been postulated due to the numerous risk factors shared by the two diseases, but has only recently been convincingly demonstrated epidemiologically 23, 24. Our genetic study indicates that endometriosis and endometrial cancer have a moderate, but significant, shared genetic etiology. Genetic correlation analyses indicated the presence of pleiotropic SNPs as well as correlation in the direction of genetic effects, particularly amongst SNPs marginally and nominally associated with each disease individually. This is consistent with our hypothesis that a proportion of endometrial cancer cases (but certainly not all) will share genetic predisposition factors in common with endometriosis cases.

Cross‐disease meta‐analysis identified one genomewide significant locus associated with the risk of developing both diseases, and several other loci worthy of prioritization for future studies. These findings indicate that genetic factors underlie at least part of the shared disease risk implied by the epidemiological evidence. The SNP most significantly associated with disease in the endometriosis–endometrial cancer meta‐analysis was rs2475335 (*P *=* *4.9 × 10^−8^). Located on chromosome 9p23, rs2475335 lies within intron 2 of an alternative transcript of the protein tyrosine phosphatase receptor type D (*PTPRD*) gene. *PTPRD* is a member of the receptor protein tyrosine phosphatase (PTP) family, a number of which have been found to function as either tumor suppressors or as oncogenes 37. *PTPRD* deletions and mutations have been detected in numerous tumor types, including endometrial tumors 38: the Catalogue of Somatic Mutations in Cancer (COSMIC) database (http://cancer.sanger.ac.uk/cosmic; accessed 10/12/2016) indicates ~5% of endometrioid carcinomas harbor *PTPRD* mutations. Mutations in *PTPRD* enhance cell growth and migration in melanoma cell lines, while the presence of mutated PTPRD protein enhanced growth and abrogated dephosphorylation of the STAT3 oncoprotein in human astrocytes 38, 39. Elevated STAT3 expression has been implicated in both endometriosis and endometrial cancer 40, 41 and has been suggested as a potential target for treatment for both diseases 42, 43. While *PTPRD* is an attractive candidate gene for regulation by rs2475335, the gene targeted by this SNP (or equally by another SNP/s in high linkage disequilibrium with rs2475335) is as yet unknown, and further experimental studies in both endometriosis and endometrial cancer models are now required to investigate the biology underlying the increased risks of both diseases associated with this variant 44.

A number of the remaining risk loci prioritized by the meta‐analysis harbor candidate genes that are potentially relevant candidates for etiology and/or treatment of endometriosis and endometrial cancer. For example, the missense variant rs2278868 located on chromosome 17q21.32 within exon 7 of the *SKAP1* gene is in perfect linkage disequilibrium (*r* ^2^ **= **1) with rs1452666, which we have previously reported as having borderline GWAS significant association with endometrial cancer in the combined GWAS and iCOGS datasets 9. SNP variation in the *SKAP1* region is associated with ovarian cancer, subtypes of which are clearly linked epidemiologically and genetically to endometriosis 25, 45 and to endometrial cancer 46, although rs2278868 is in extremely low LD with the top ovarian cancer SNP rs9303542 (*r* ^*2*^ **= **0.034) 33. Publically available gene expression data (http://www.gtexportal.org ) indicate the potential for SNPs rs2278868 (and rs1452666) and rs9303542 to act as expression quantitative trait loci (eQTLs) for *SKAP1*, altering *SKAP1* expression in liver, tibial nerve, testis, and pancreatic tissues, as well as for additional neighboring genes (e.g., *SNX11*,* HOXB2,* and *HOXB3*) in various tissues.

Other SNPs of interest include rs12303900 on chromosome 12q21, located between the *KITLG* and *DUSP6* genes. *DUSP6* is a critical regulator of ERK signaling, a pathway dysregulated in both endometriosis and endometrial cancer and a potential target for treatment for both diseases 47, 48, 49. SNP rs10008492, located on chromosome 4p14, is an eQTL for nearby toll‐like receptors *TLR1* and *TLR6* (http://www.gtexportal.org). Both *TLR1* and *TLR6* are upregulated in endometriotic mesenchymal stem cells 50 and are expressed in endometrial cancer cell lines 51. However, as for the *PTPRD* locus, all of these association results need to be further validated in additional replication datasets for both diseases, and relevant functional studies undertaken, before more is hypothesized about their genetic and biological effects on the risk of both endometriosis and endometrial cancer.

In this cross‐disease genetic correlation and genomewide association study, we have provided evidence for overlap in genetic risk factors for endometriosis and endometrial cancer. Our genetic correlation analysis supports recent large epidemiological studies indicating an increased risk of endometrial cancer in women previously diagnosed with endometriosis, while the cross‐disease meta‐analysis has revealed plausible loci that could increase the risk of both diseases and which should be pursued further in functional studies. This work on endometriosis and endometrial cancer also adds further evidence to the utility of cross‐disease genetic correlation and GWAS analyses as tractable and attractive methodologies to identify susceptibility loci that predispose to multiple diseases, which could lead to new diagnostic or treatment options for affected individuals.

## Conflict of Interest

None declared.

## Supporting information


**Figure S1.** Forest plots of association between the top 13 SNPs in the endometriosis‐endometrial cancer meta‐analysis and each of the datasets included in the analysis.

**Table S1.** Results for the top SNPs from the endometriosis and endometrial cancer (ANECS‐SEARCH and NSECG) GWAS meta‐analysis, with iCOGS as the endometrial cancer replication dataset.
