# Supplementary material for: Genetic overlap between endometriosis and endometrial cancer: evidence from cross‐disease genetic correlation and GWAS meta‐analyses
Source: Cancer Med. 2018 Apr 2;7(5):1978–87. doi: 10.1002/cam4.1445 (PMC5943470; doi:10.1002/cam4.1445)
Supplement: Supplementary file 1 — Figure S1. Forest plots of association between the top 13 SNPs in the endometriosis‐endometrial cancer meta‐analysis and each of the datasets included in the analysis. [file CAM4-7-1978-s001.pdf]

Supplementary Figure 1. Forest plots of association between the top 13 SNPs in the endometriosis-endometrial cancer meta-analysis and each of the datasets included in the analysis. Effect sizes (odds ratios) are shown as squares for the individual datasets, and as circles for the two meta-analyses ("GWAS only" (including the endometriosis and endometrial cancer GWAS datasets), and "GWAS + endometrial cancer replication" (including all GWAS datasets and the iCOGS endometrial cancer replication dataset)).

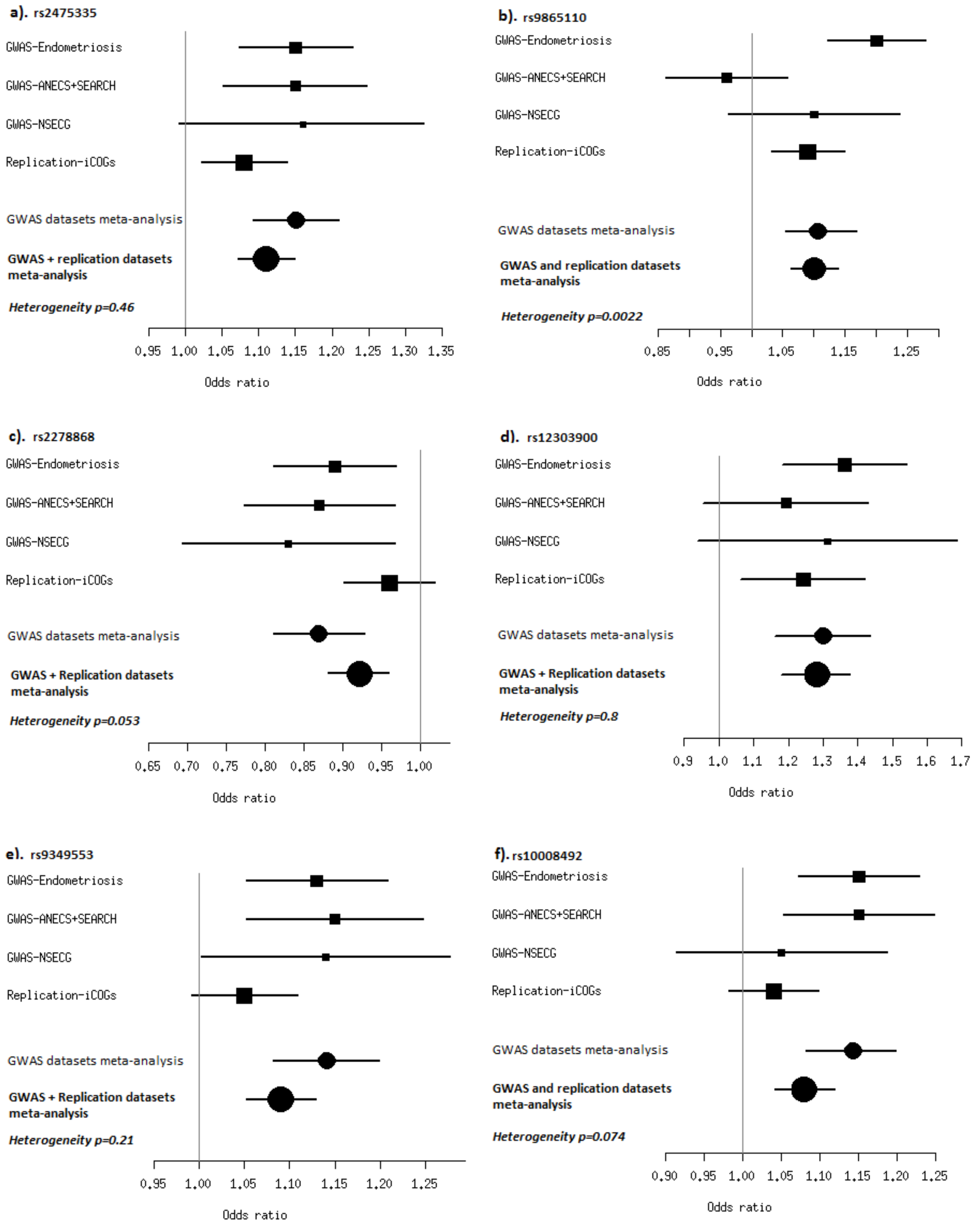

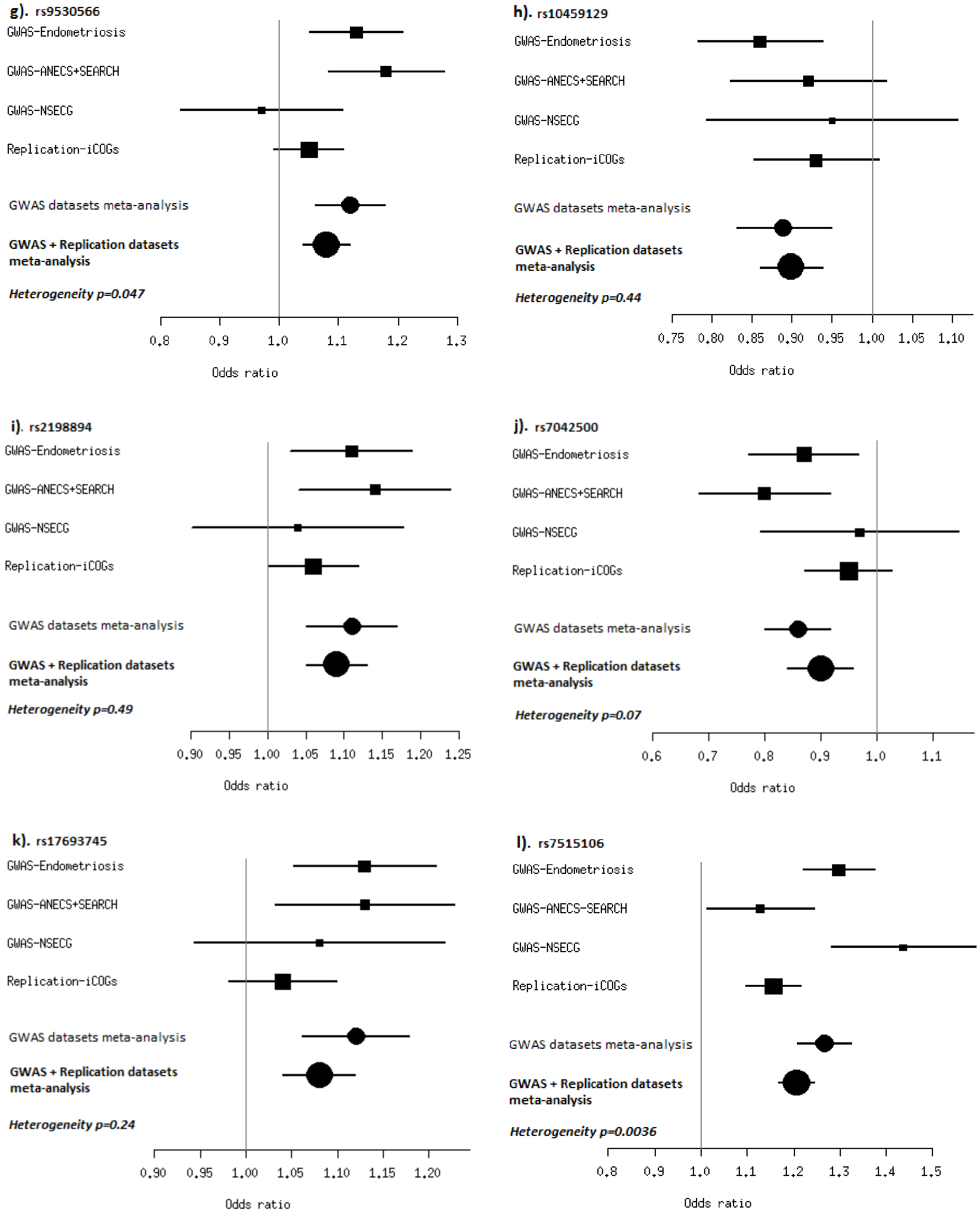
g). rs9530566
GWAS-Endometriosis
GWAS-ANECS+SEARCH
GWAS-NSECG
Replication-iCOGs
GWAS datasets meta-analysis
GWAS + Replication datasets meta-analysis
Heterogeneity p=0.047
0.8 0.9 1.0 1.1 1.2 1.3
Odds ratio
h). rs10459129
GWAS-Endometriosis
GWAS-ANECS+SEARCH
GWAS-NSECG
Replication-iCOGs
GWAS datasets meta-analysis
GWAS + Replication datasets meta-analysis
Heterogeneity p=0.44
0.75 0.80 0.85 0.90 0.95 1.00 1.05 1.10
Odds ratio
i). rs2198894
GWAS-Endometriosis
GWAS-ANECS+SEARCH
GWAS-NSECG
Replication-iCOGs
GWAS datasets meta-analysis
GWAS + Replication datasets meta-analysis
Heterogeneity p=0.49
0.90 0.95 1.00 1.05 1.10 1.15 1.20 1.25
Odds ratio
j). rs7042500
GWAS-Endometriosis
GWAS-ANECS+SEARCH
GWAS-NSECG
Replication-iCOGs
GWAS datasets meta-analysis
GWAS + Replication datasets meta-analysis
Heterogeneity p=0.07
0.6 0.7 0.8 0.9 1.0 1.1
Odds ratio
k). rs17693745
GWAS-Endometriosis
GWAS-ANECS+SEARCH
GWAS-NSECG
Replication-iCOGs
GWAS datasets meta-analysis
GWAS + Replication datasets meta-analysis
Heterogeneity p=0.24
0.90 0.95 1.00 1.05 1.10 1.15 1.20
Odds ratio
l). rs7515106
GWAS-Endometriosis
GWAS-ANECS-SEARCH
GWAS-NSECG
Replication-iCOGs
GWAS datasets meta-analysis
GWAS + Replication datasets meta-analysis
Heterogeneity p=0.0036
0.8 0.9 1.0 1.1 1.2 1.3 1.4 1.5
Odds ratio

m). rs1755833

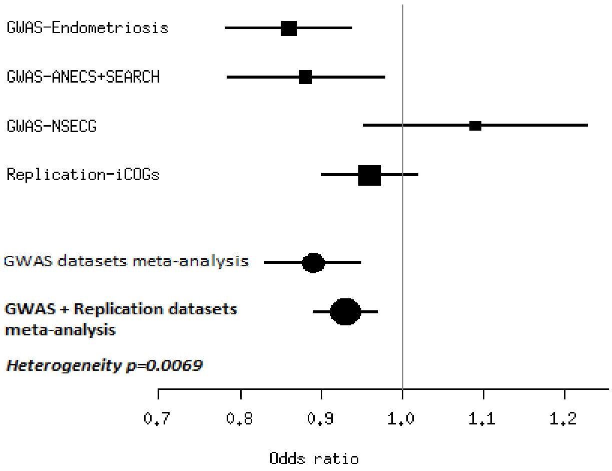
GWAS-Endometriosis
GWAS-ANECS+SEARCH
GWAS-NSECG
Replication-iCOGs
GWAS datasets meta-analysis
GWAS + Replication datasets meta-analysis
Heterogeneity p=0.0069
0.7
0.8
0.9
1.0
1.1
1.2
Odds ratio
